# Inference of the Activity Timeline of Cattle Foraging on a Mediterranean Woodland Using GPS and Pedometry

**DOI:** 10.3390/s110100362

**Published:** 2010-12-31

**Authors:** Eugene D. Ungar, Iris Schoenbaum, Zalmen Henkin, Amit Dolev, Yehuda Yehuda, Arieh Brosh

**Affiliations:** 1 Department of Agronomy and Natural Resources, Institute of Plant Sciences, Agricultural Research Organization—the Volcani Center, P.O. Box 6, Bet Dagan 50250, Israel; 2 The Robert H. Smith Institute for Plant Sciences and Genetics in Agriculture, Faculty of Agriculture, Food and Environment, Hebrew University of Jerusalem, Rehovot 76100, Israel; E-Mail: isi_shin@yahoo.com; 3 Beef Cattle Section, Newe Ya’ar Regional Research Center, Agricultural Research Organization, P.O. Box 1021, Ramat Yishay 30095, Israel; E-Mail: henkinz@volcani.agri.gov.il (Z.H.); brosha@volcani.agri.gov.il (A.B.); 4 Migal–Galilee Technology Center, P.O. Box 831, Kiryat Shmona 11016, Israel; E-Mail: amit.dolev12@gmail.com

**Keywords:** calibration, discriminant analysis, partition analysis, grazing behavior, classification, GPS collar, motion sensors, pedometer, step count

## Abstract

The advent of the Global Positioning System (GPS) has transformed our ability to track livestock on rangelands. However, GPS data use would be greatly enhanced if we could also infer the activity timeline of an animal. We tested how well animal activity could be inferred from data provided by Lotek GPS collars, alone or in conjunction with IceRobotics IceTag pedometers. The collars provide motion and head position data, as well as location. The pedometers count steps, measure activity levels, and differentiate between standing and lying positions. We gathered synchronized data at 5-min resolution, from GPS collars, pedometers, and human observers, for free-grazing cattle (n = 9) at the Hatal Research Station in northern Israel. Equations for inferring activity during 5-min intervals (n = 1,475), classified as Graze, Rest (or Lie and Stand separately), and Travel were derived by discriminant and partition (classification tree) analysis of data from each device separately and from both together. When activity was classified as Graze, Rest and Travel, the lowest overall misclassification rate (10%) was obtained when data from both devices together were subjected to partition analysis; separate misclassification rates were 8, 12, and 3% for Graze, Rest and Travel, respectively. When Rest was subdivided into Lie and Stand, the lowest overall misclassification rate (10%) was again obtained when data from both devices together were subjected to partition analysis; misclassification rates were 6, 1, 26, and 17% for Graze, Lie, Stand, and Travel, respectively. The primary problem was confusion between Rest (or Stand) and Graze. Overall, the combination of Lotek GPS collars with IceRobotics IceTag pedometers was found superior to either device alone in inferring animal activity.

## 1. Introduction

A universal feature of extensive rangelands is high spatial heterogeneity of their utilization by livestock [1–3]. This heterogeneity may derive from features of the landscape, such as topography, forage availability and quality [4–6], from features of the management system, such as herding versus free-ranging [7], and from the placement of watering [8,9] and supplementation points [10]. Because of this heterogeneity, expression of animal density as the quotient of the total number of animals on a site divided by its area has limited biological validity, and is a poor predictor of landscape processes such as degradation and desertification [11,12]. An understanding of the impact of livestock on the landscape requires spatially explicit study of its utilization.

Historically it has been very difficult to study the spatial component of landscape use by animals, but application of the Global Positioning System (GPS) to the study of grazing systems has led to a quantum leap in our ability to track many species of livestock and wildlife [13]. The GPS is now commonly employed in range management research, to monitor and analyze the use of areas by livestock, either solely [14,15] or in combination with wildlife [16]. Animal-borne GPS devices provide continuous and accurate records of animal location over time. However, location alone does not represent a complete picture with regard to estimation of the spatial distribution of grazing pressure, because animals do not graze actively all the time; they divide their time among several activities, such as resting, traveling (without grazing), and active grazing. It would be a great enhancement of GPS data use if we could also infer the activity timeline of an animal.

The inference of behavior from data provided by sensors mounted in the head region, such as a tri-axial accelerometer for goats [17] and a pitch and roll tilt sensor for sheep [18], has been explored. Some GPS devices incorporate sensors that can give an indication of activity. Lotek GPS collars (Lotek Wireless Inc., Newmarket, ON, Canada) include sensors of motion along two axes, and these store the numbers of movements they register during each GPS-fix interval. These data, in conjunction with the distances between consecutive GPS locations, have been used in a study of calibration of statistical models for inferring animal activity, but the rate of misclassification in that study was found to be significant [19]. One might expect the distance between GPS locations in itself to be an adequate indicator of activity at three levels–low, medium, and high—which would correspond to resting, grazing, and walking, respectively. However, whereas this works fairly well for walking, GPS error and the fact that the distance between consecutive GPS location readings of a stationary device is not zero, blurs the distinction between resting and grazing [20]. The addition of information from motion sensors does improve matters, but not as much as might be expected: first, resting animals move their heads; second, the precise fit of the GPS collar around the neck may have a significant influence on motion sensor responses and counts [21]; and third, we suspect that device sensitivity differs among factory batches. Visual calibration of individual cow-collar combinations or even of individual collars is not a practicable option, because visual observations are extremely time consuming.

We hypothesized that leg movements might correspond to activity more directly and mechanistically than head and neck movements. Combining data from a pedometer with those from a GPS collar might, therefore, enable reduction in the rates of misclassification of animal activity. This would, however, require a pedometer of exceptional temporal resolution. In recent years, such a pedometer has become available in the form of the IceTag (IceRobotics, Scotland, UK). This device provides a step count and a time allocation between three states (lying, standing, active) for any time resolution down to 1 s. The distinction between lying and standing is an added benefit in the context of studies that integrate the spatial dimension of landscape use with calculations of energy expenditure [22,23].

The purpose of the present study was to examine whether the addition of this sophisticated pedometer would enable us to reduce the rates of misclassification of animal activity. The overall approach was to gather synchronized GPS collar, pedometer and observer data for free-grazing cattle, to derive calibration equations for prediction of activity on the basis of data from each device separately and from both together, and to compare the prediction accuracy of these equations.

## 2. Experimental Section

### 2.1. Study Site

The study was conducted at the 280-ha Hatal Research Station, located 15 km east of the Mediterranean coastline in western Galilee, Israel (35°15′E, 33°01′N; alt. 200–500 m a.s.l.). The climate is typically Mediterranean, with mild, wet winters and hot, dry summers. Average annual precipitation is 780 mm, which falls almost entirely from November through March. The area consists of moderate to steep slopes, with a 20–40% cover of limestone and dolomite rocks, between which there are pockets of terra rossa soil up to 40 cm deep. The vegetation is dominated by scrub-oak woodland (*Quercus calliprinos*), interspersed with batha vegetation comprising shrubs and dwarf shrubs, mainly *Calicotome villosa* and *Sarcopoterium spinosum* [24]. Herbaceous vegetation grows in patches interspersed among the woody vegetation, and provides high-quality forage during 3–4 months in the spring. The station is populated by a beef suckler herd of approximately 100 cows that remain on the station throughout the year. The cows are local Baladi × Brahman or Hereford crossbreds of 455 kg mean live weight. Diets comprise a mix of woody and herbaceous vegetation, and supplementation is provided in the late summer and autumn [25].

### 2.2. Observations of Cow Activity

Observations of cow activity were conducted in a 1.5-ha fenced observation plot, using animals drawn from the above herd. The herd is familiar with this area, which is part of a larger plot in which the herd is kept during the grazing deferment period at the beginning of each growing season. In May 2006, a group of eight cows were brought into the observation plot for a 4-day period after four of the animals had been fitted with GPS and pedometer devices (described below). Observations of these four animals were conducted on the second, third and fourth days, after which the devices were removed from the animals and the entire group rejoined the herd. A similar procedure was followed in November 2006 with eight other cows, of which five were fitted with GPS and pedometer devices; these animals spent 3 days in the observation plot, and observations were conducted during the last two days.

On the first two observation days in May and the first observation day in November, two observers conducted observations from 0600 till 1700, and on the last observation day of each season they conducted observations from 0630 till 0830 (May) and from 0600 till 1000 (November). The observers coordinated their observations to ensure that all of the four (May) or five (November) animals wearing the sensors were being tracked at all times. Each observer was able to keep track of the activity of up to four cows simultaneously; the cows behaved as a group, in that their activities were largely synchronized. For example, lying and standing activities usually occurred in the same area of the paddock, so it was possible to observe the whole group simultaneously. The observers moved through the plot in a quiet and unobtrusive way so as not to disturb animal behavior, and the cows were quite indifferent to the presence of the observers, so that it was possible to get as close as 3 m without disturbing their behavior. Such close distances were sometimes required because of the presence of trees and tall bushes.

The timepiece of each observer was synchronized to an accurate clock at the beginning of each observation day. When visual observations of activity commenced, the observer recorded the date, the identity of the cow, the time (hh:mm:ss), and the activity. Activity was defined as Graze, Lie, Stand (without grazing) or Travel (walking without grazing). A new data record, comprising the time and activity, was added provisionally as soon as a cow switched to a different activity, and the transition was confirmed if the new activity continued for at least 30 s. A transition to Unknown activity was recorded for as long as a cow’s activity could not be observed clearly.

### 2.3. GPS Collars

The observed animals wore 3300LR series Lotek GPS collars. The collars can integrate and store a GPS fix at user-determined intervals, together with information from on-board temperature (ambient) and motion sensors. The collars contain two tilt switches to sense motion; they are sensitive to acceleration and deceleration, as well as tilting. The sensors are mounted in the collar at right angles to one another, with the long axes parallel to the ground. One sensor is most sensitive to left–right, and the other to fore-aft movement.

The motion sensors acquire data within a cyclic activity-sampling period. The parameters for the activity-sampling period and the GPS fix interval, as well as the mode of operation of the motion sensors, are set by the user via a software interface. The GPS fix interval can range from 5 to 360 min, and the activity-sampling period can be set between 1 and 60 min (but not greater than the GPS fix interval–1 min). The activity counters tally up to a maximum number of 255 counts. At the start of a GPS fix, the numbers of motion sensor “hits” in the left–right and fore–aft directions that were recorded since the previous activity-sampling are stored, and the system is reinitialized. Activity count data can be stored either as left–right and fore–aft counts since the previous completed activity-sampling period, or as the mean count numbers from several activity-sampling periods since the start of the previous GPS fix. In the latter case, the number of such periods = integer (GPS fix interval–1 min) / activity-sampling period.

The collar also contains a head-down activity sensor that consists of a switch that opens or closes according to the head position. The contact closes and the collar registers a down position when the collar is at an angle >7.5° clockwise to perpendicular to the horizontal plane. The contact is open when the collar angle is >7.5° anticlockwise. In the intermediate angle range of ±7.5° to perpendicular, the contact could be open or closed. The collar stores the percentage of time the sensor registers the down position during an activity sampling period.

The collars were configured to integrate a GPS location every 5 min and to store motion-sensor counts for the interval between GPS fixes (strictly—for the first 4 min of the 5-min interval between GPS fixes). Prior to deployment on the observed animals, the internal clock of each collar was synchronized against an accurate clock. On retrieval of the collars from the animals, the coordinates of recorded positional fixes (as degrees latitude and longitude) and accompanying data were downloaded to a computer. Accompanying data included: elevation, date, time, ambient temperature, left–right motion sensor count (Lotek_LeftRight_; value between 0 and 255), fore–aft motion sensor count (Lotek_ForAft_; value between 0 and 255), percentage of time when the head-angle sensor registered the down position (Lotek_HeadDown_; value between 0 and 100), and satellite-related information. Coordinates were stored in Solved mode, which does not permit subsequent differential correction of the data. In general, a long collar-deployment time is used at the study site because of the great difficulty in corralling the herd from among the thickly wooded landscape, and this mandated storage in Solved mode because of memory constraints. Coordinates were converted from UTM WGS84 to Israel Transverse Mercator using ArcGIS 9.X (ESRI, USA), and the straight-line distance between consecutive GPS coordinates at 5-min intervals was computed (Lotek_Distance_; m).

### 2.4. Pedometers

The observed cows wore an IceTag pedometer on their rear right leg, centered approximately 15 cm above the fetlock joint. The IceTag uses accelerometer technology to analyze the movement of an animal’s leg, and classifies its activity as lying (the animal is lying down), standing (the animal is standing still), or active (the animal is standing and moving). Where the animal is active, a step count indicates the number of steps taken by the animal, *i.e.*, by the leg carrying the tag. The device stores data at a time resolution of 1 s, and the data can be aggregated to any lower resolution with the download software. For the present analysis, values were aggregated over 1-min intervals. Relevant data fields were: date and time; percentage of time interval spent lying (IceTag_Lying_); percentage of time interval spent standing (IceTag_Standing_); percentage of time interval spent active (IceTag_Active_); number of steps taken during time interval (IceTag_Steps_). Prior to deployment, the internal clock of each pedometer was synchronized against an accurate clock.

### 2.5. Analysis

For each cow observed, data from the three sources—Lotek GPS collar, IceRobotics IceTag pedometer, visual observation—were merged into a single data file as follows. First, GPS collar data were entered, *i.e.*, the motion sensor counts and GPS distance corresponding to the 5 min preceding the time value given on each record. Then, five time columns were added, one for each of the preceding five minutes. For each of these time columns, the corresponding record in the pedometer data file was located, the four pedometer variables (IceTag_Lying_, IceTag_Standing_, IceTag_Active_, IceTag_Steps_) were retrieved, and 5-min averages were computed for each pedometer variable. A similar procedure was followed for the visual observation data: for each of the five time columns defined above, the corresponding record in the observation data file was located, and the activity (Graze, Lie, Stand, Travel, and Unknown) was retrieved.

The representative activity for a 5-min period was defined according to two classifications of activity. In the first classification, which we refer to as GRT (Graze, Rest, Travel), Lie and Stand were merged as Rest, and activity was set to whichever of Graze, Rest or Travel accounted for at least four of the 5 min; if none occupied 4 min the activity was classed as Mixed. In the second classification, which we refer to as GLST, the distinction between Lie and Stand was retained, and activity was set to whichever of Graze, Lie, Stand or Travel accounted for at least four of the 5 min, or to Mixed if none did so.

The main analytical methods were based on discriminant analysis and partition analysis (classification tree) applied by means of the JMP software, version 7.0.2 (SAS Institute Inc., Cary, NC, USA). Separate analyses were conducted for the GRT and GLST classifications of activity, based on GPS collar data only, pedometer data only, and data from both devices. For discriminant analysis, the linear common covariance method was used. For partition analysis, the maximized significance criterion was used with a minimum split size of 5, although the minimum cell count in any analysis was >30. We used K-fold cross-validation with K = 5 to test model robustness and to avoid over-fitting. Both analytical methods were evaluated in terms of the overall misclassification rate and the misclassification rate for each activity. Data from both seasons of data collection were pooled for all analyses. A much more extensive data set, distributed over an entire annual cycle, would be needed for rigorous examination of seasonal effects on the calibration equations.

The combination of three device configurations, two classifications of activity, and two analytic methods generates 12 analyses. Results of the six analyses for the simpler (GRT) classification of activity are presented first, with results based on GPS collar data only, pedometer data only, and data from both devices being presented in that order. Within each of these, results for discriminant analysis are presented before those for partition analysis. Results of the six analyses for the more detailed (GLST) classification of activity are then presented in the same order.

## 3. Results

### 3.1. Total Observation Time and Allocation among Activities

A total of 133.5 h of activity classifiable as GRT or GLST, *i.e.*, excluding Unknown, was collected. Mean total classifiable observation time per cow was 18.9 h in May and 11.6 h in October. In both seasons the animals spent a proportion of 0.59 of the observation time in resting. The proportions of observation time spent in grazing were 0.31 in May and 0.36 in October; those in traveling were 0.10 and 0.05, respectively; those in lying were 0.42 and 0.20, respectively; and those in standing were 0.17 and 0.39, respectively.

### 3.2. Mixed Periods

The appropriateness of using a 5-min time resolution to define activity depends on the proportion of such time intervals that is entirely or largely devoted to one activity. We found that 87.1% of 5-min intervals contained one activity only, and in a further 7.6% of the 5-min intervals 4 min were devoted to one activity. Thus, only 5.3% of the intervals were spent in “mixed” activities, and these were excluded from the calibration analysis. The remaining number of 5-min periods analyzed was 1,475.

### 3.3. Overview of Device Responses

**Figure 1.**
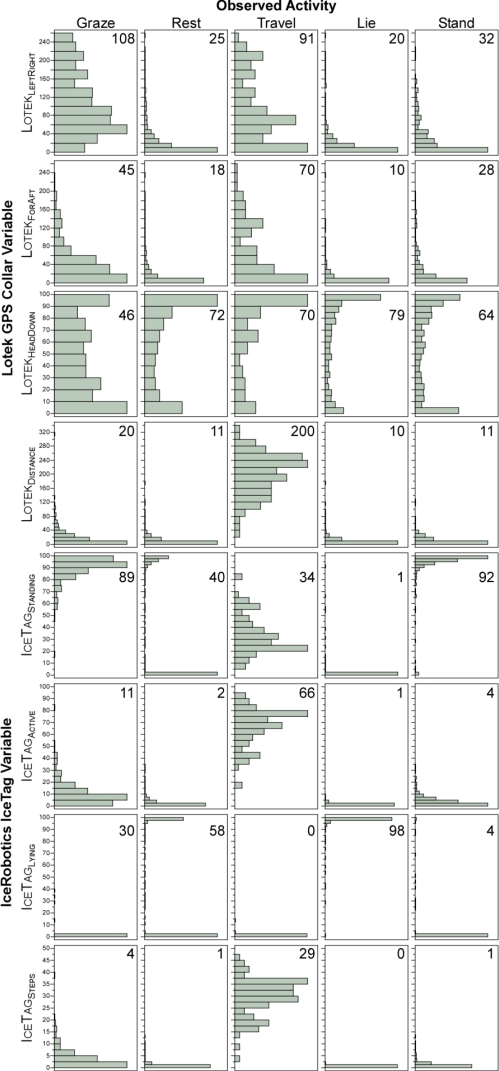
Frequency distributions of Lotek GPS collar and IceRobotics IceTag pedometer readings according to activity category. Graze, Rest and Travel results are based on 1,475 five-minute intervals; Stand and Lie results are based on 1,463 five-minute observations. Number in each panel is the mean.

In order to visualize how well the four Lotek GPS collar variables and four IceRobotics IceTag pedometer variables might serve to classify animal activity, we examined their means and frequency distributions according to activity (Figure 1). Activity (GRT) was highly significant (*P* < 0.001) in the non-parametric analysis (Kruskal-Wallis) of each of these eight variables.

When the activity was defined as Graze, the Lotek collar left-right motion-sensor count (Lotek_LeftRight_) for a 5-min interval ranged from 0 to 255 with a peak in the distribution in the region of 40 to 60, and a mean value of 108. Travel also yielded a diffuse frequency distribution, although with a greater proportion of values in the low range, and a mean value of 91. Rest yielded a highly skewed frequency distribution, with 75% of values being ≤ 30 and 13% being exactly 0 but, nevertheless, the entire range of possible values was obtained. For Rest the mean value of Lotek_LeftRight_ was 25; and subdivision into Stand and Lie yielded similar results, although Lie yielded a more strongly skewed distribution, with 75% quantile values of 20 and 44 for Lie and Stand, respectively.

For all activities other than Graze, the Lotek collar fore-aft motion sensor count (Lotek_ForAft_) yielded similar frequency distributions to those obtained for left-right motion (Lotek_LeftRight_), but more strongly skewed toward low values; no activity yielded predominantly high Lotek_ForAft_ values. For Rest, 75% of values were ≤19 and 28% of values were exactly 0; for the Lie subdivision of Rest the 75% quantile was 10, and 35% of values were exactly 0. However, low or zero values were not uncommon also for Graze and Travel. Correlation coefficients between Lotek_LeftRight_ and Lotek_ForAft_ were very low: 0.0192, 0.0775, and −0.1746 for Graze, Rest and Travel, respectively.

The Lotek collar head-down sensor (Lotek_HeadDown_) yielded a broadly U-shaped frequency distribution for all activities, and its values did not correlate well with those of either Lotek_LeftRight_ or Lotek_ForAft_. The straight-line distance between successive Lotek collar GPS locations (Lotek_Distance_) ranged from 0.08 to 338 m. When the activity was defined as Graze, 90% of the values were ≤50 m. The frequency distribution of Lotek_Distance_ was most highly skewed for Rest activity, with 90% of values being ≤23 m. Travel yielded a frequency distribution that peaked at approximately 230–240 m, with the 2.5% quantile at 56 m. Lie and Stand yielded very similar frequency distributions to that of Rest.

**Figure 2.**
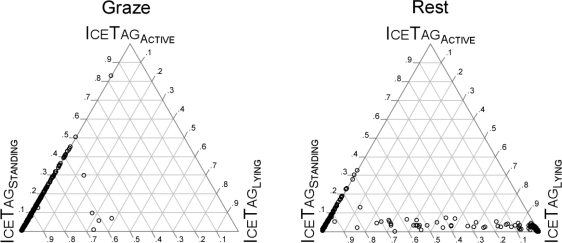

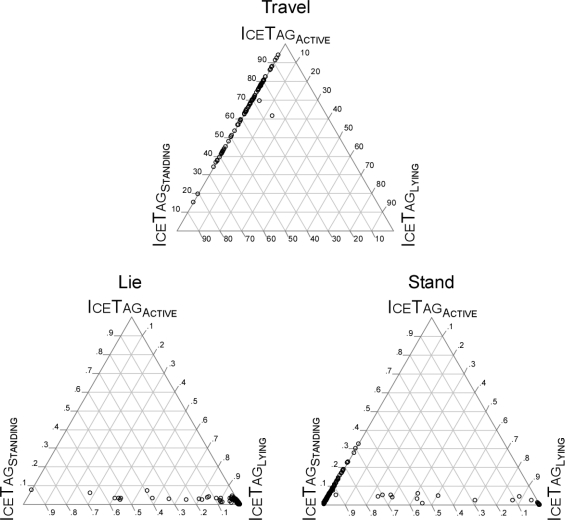
Ternary plot (unit-sum triangle) of the proportion of a 5-min interval allocated to the active, standing and lying states, as defined by the IceRobotics IceTag pedometer, according to category of observed activity. Graze, Rest and Travel categories are based on 1,475 five-minute intervals. Lie and Stand categories are based on 1,463 five-minute observations. States have a proportion of 1 at the vertex of the triangle at which they are marked and 0 along the opposing edge.

The three IceRobotics variables representing the time allocation between the device-defined states of standing (IceTag_Standing_), lying (IceTag_Lying_), and active (IceTag_Active_) were examined together because they sum to 100%. Figure 2 shows these allocations as a series of ternary plots. The vast majority of points representing Graze activity fell on the IceTag_Standing_ -IceTag_Active_ axis for IceTag_Lying_ = 0%, with IceTag_Standing_ values >50%. Almost all points representing Travel also fell on this same axis, although most of them were spread broadly over the range 35% < IceTag_Active_ < 95%.

The majority of points representing Rest were distributed along an IceTag_Standing_ -IceTag_Lying_ axis of approximately IceTag_Active_ = 5%. The Lie subdivision of Rest yielded a large concentration of points near IceTag_Lying_ = 100%, and the Stand subdivision of Rest yielded a large concentration of points on the IceTag_Standing_ -IceTag_Active_ axis for IceTag_Lying_ = 0%, with IceTag_Standing_ values >80%.

The number of steps recorded by the IceRobotics pedometer (IceTag_Steps_) ranged from 0 to 47 per 5-min interval, with 23% of observations recording zero. For Graze, 90% of values were <9. For Rest, values were even more concentrated near the zero end of the distribution, with 90% of values being <2. Walk yielded an average of 29 steps, with 90% of values being >18.

### 3.4. Inference of GRT Activity from Lotek Collar Variables

Inference of GRT activity from Lotek collar variables by discriminant analysis yielded an overall misclassification rate of 16% (Table 1; Wilks’ Lambda = 0.1291, *P* < 0.001). However, the misclassification rates differed considerably among the activities: eight of the 88 cases (9%) of Travel; 89 of the 898 cases (10%) of Rest, almost all misclassified as Graze; and 139 of the 489 cases (28%) of Graze, almost all misclassified as Rest.

Partition analysis entailed trade-offs as the number of splits in the decision tree increased. After three splits (creating four partitions), the overall misclassification rate was 17%, and the separate misclassification rates for Graze, Rest and Travel were 16, 19, and 3%, respectively (Table 2). The partitions were:

1. Lotek_LeftRight_ < 39 and Lotek_Distance_ ≥ 96 m: Travel (81% probability)
2. Lotek_LeftRight_ < 39 and Lotek_Distance_ < 96 m: Rest (91% probability)
3. Lotek_LeftRight_ ≥ 39 and Lotek_Distance_ ≥ 100 m: Travel (91% probability)
4. Lotek_LeftRight_ ≥ 39 and Lotek_Distance_ < 100 m: Graze (71% probability)

Introducing an additional split reduced the overall misclassification rate to 14%, but the misclassification rates for Graze, Rest and Travel became 33, 5, and 3%, respectively (Table 2). The first four partitions were as above, to which were added:

5. Lotek_LeftRight_ ≥ 39 and Lotek_Distance_ < 100 m and Lotek_HeadDown_ ≥ 77%: Rest (60% probability)
6. Lotek_LeftRight_ ≥ 39 and Lotek_Distance_ < 100 m and Lotek_HeadDown_ < 77%: Graze (90% probability)

### 3.5. Inference of GRT Activity from IceRobotics Pedometer Variables

Inference of GRT activity by discriminant analysis of IceRobotics pedometer outputs yielded an overall misclassification rate of 26% (Table 1; Wilks’ Lambda = 0.1283, *P* < 0.001). Misclassification rates separated according to activity were 2, 40, and 7% for Graze, Rest, and Travel, respectively. The large error rate for Rest derived from misclassification as Graze. Partition analysis on the basis of IceTag_Active_ only yielded an overall misclassification rate of 18%, and misclassification rates separated according to activity of 12, 22, and 3% for Graze, Rest, and Travel, respectively (Table 2). The partitions were:

1. IceTag_Active_ < 3%: Rest (94% probability)
2. IceTag_Active_ ≥ 3% and IceTag_Active_ < 37%: Graze (68% probability)
3. IceTag_Active_ ≥ 37%: Travel (87% probability)

After five splits the overall misclassification rate was 15%, and the misclassification rates separated for Graze, Rest, and Travel were 12, 18, and 3%, respectively (Table 2). The partitions were:

1. IceTag_Active_ < 3% and IceTag_Standing_ < 66%: Rest (100% probability)
2. IceTag_Active_ < 3% and IceTag_Standing_ ≥ 66%: Rest (81% probability)
3. IceTag_Active_ ≥ 3% and IceTag_Active_ ≥ 37%: Travel (87% probability) [reduces to IceTag_Active_ ≥ 37%]
4. IceTag_Active_ ≥ 3% and IceTag_Active_ < 37% and IceTag_Steps_ < 2.4 and IceTag_Standing_ < 89%: Rest (97% probability)
5. IceTag_Active_ ≥ 3% and IceTag_Active_ < 37% and IceTag_Steps_ < 2.4 and IceTag_Standing_ ≥ 89%: Graze (57% probability)
6. IceTag_Active_ ≥ 3% and IceTag_Active_ < 37% and IceTag_Steps_ ≥ 2.4: Graze (85% probability)

### 3.6. Inference of GRT Activity from Lotek Collar and IceRobotics Pedometer Outputs

Inference of GRT activity by discriminant analysis of both Lotek collar and IceRobotics pedometer outputs yielded an overall misclassification rate of 11% (Table 1; Wilks’ Lambda = 0.0859, *P* < 0.001). Misclassification rates separated according to activity were fairly evenly balanced, at 11, 11, and 6% for Graze, Rest, and Travel, respectively. Partition analysis with two splits yielded an overall misclassification rate of 17% and separate rates of 11, 22, and 3% for Graze, Rest, and Travel, respectively (Table 2). The partitions were:

1. IceTag_Active_ < 3%: Rest (94% probability)
2. IceTag_Active_ ≥ 3% and Lotek_Distance_ ≥ 96 m: Travel (91% probability)
3. IceTag_Active_ ≥ 3% and Lotek_Distance_ < 96 m: Graze (68% probability)

Increasing the number of splits to six (involving IceTag_Active_, Lotek_LeftRight_, Lotek_HeadDown_ and Lotek_Distance_) resulted in an overall misclassification rate of 10% and separate rates of 8, 12, and 3% for Graze, Rest, and Travel, respectively (Table 2). The partitions were:

1. IceTag_Active_ < 3% and Lotek_LeftRight_ < 52: Rest (99% probability)
2. IceTag_Active_ < 3% and Lotek_LeftRight_ > 52 and Lotek_HeadDown_ ≥ 85%: Rest (96% probability)
3. IceTag_Active_ < 3% and Lotek_LeftRight_ > 52 and Lotek_HeadDown_ < 85%: Graze (82% probability)
4. IceTag_Active_ ≥ 3% and Lotek_Distance_ ≥ 96 m: Travel (91% probability)
5. IceTag_Active_ ≥ 3% and Lotek_Distance_ < 96 m and Lotek_LeftRight_ < 48 and IceTag_Active_ < 7%: Rest (81% probability)
6. IceTag_Active_ ≥ 3% and Lotek_Distance_ < 96 m and Lotek_LeftRight_ < 48 and IceTag_Active_ ≥ 7%: Graze (66% probability)
7. IceTag_Active_ ≥ 3% and Lotek_Distance_ < 96 m and IceTag_Active_ ≥ 48%: Graze (84% probability)

### 3.7. Inference of GLST Activity from Lotek Collar Outputs

The inference of GLST activity by discriminant analysis of Lotek collar outputs yielded an overall misclassification rate of 36% (Table 1; Wilks’ Lambda = 0.1214, *P* < 0.001). The misclassification rate of Graze as Lie was relatively low (9%), but that as Stand was relatively high (22%). Lie and Stand were frequently confused: 26% of Lie intervals were misclassified as Stand, and 41% of Stand intervals were misclassified as Lie. The overall misclassification rate for Stand reached 54%. Partition analysis failed to classify any intervals as Stand within the first four splits. In contrast, the misclassification rates for Graze were 15% (split 1) and 16% (splits 2–4), those for Lie were 11% (splits 1 and 2) and 12% (splits 3 and 4), and those for Travel were 100% (split 1), 32% (split 2), and 3% (splits 3 and 4). By the sixth split, the misclassification rate for Stand fell to 56%, but this was at the expense of a large increase in the misclassification rate for Lie (from 12% to 35%). At the sixth split, the overall misclassification rate was 32%, and those for Graze and Travel were 16 and 3%, respectively (Table 2). The partitions were:

1. Lotek_LeftRight_ < 39 and Lotek_Distance_ ≥ 96 m: Travel (81% probability)
2. Lotek_LeftRight_ < 39 and Lotek_Distance_ < 96 m and Lotek_ForAft_ < 9 and Lotek_HeadDown_ < 1%: Stand (80% probability)
3. Lotek_LeftRight_ < 39 and Lotek_Distance_ < 96 m and Lotek_ForAft_ < 9 and Lotek_HeadDown_ ≥ 1%: Lie (74% probability)
4. Lotek_LeftRight_ < 39 and Lotek_Distance_ < 96 m and Lotek_ForAft_ ≥ 9: Stand (44% probability)
5. Lotek_LeftRight_ ≥ 39 and Lotek_Distance_ ≥ 100 m: Travel (91% probability)
6. Lotek_LeftRight_ ≥ 39 and Lotek_Distance_ < 100 m and Lotek_HeadDown_ ≥ 77%: Graze (39% probability)
7. Lotek_LeftRight_ ≥ 39 and Lotek_Distance_ < 100 m and Lotek_HeadDown_ < 77%: Graze (90% probability)

### 3.8. Inference of GLST Activity from IceRobotics Pedometer Outputs

Inference of GLST activity from discriminant analysis of IceRobotics pedometer outputs yielded an overall misclassification rate of 21% (Table 1; Wilks’ Lambda = 0.0100, *P* < 0.001). Misclassification rates according to activity were 45, 1, 19, and 7% for Graze, Lie, Stand, and Travel, respectively. The percentage of Stand intervals that were misclassified as Graze was 16%, but 43% of Graze intervals were misclassified as Stand. After three splits partition analysis yielded an overall misclassification rate of 19%, and rates separated according to activity were 37, 1, 20, and 17% for Graze, Lie, Stand, and Travel, respectively (Table 2). The partitions were:

1. IceTag_Standing_ < 55% and IceTag_Active_ < 48%: Lie (97% probability)
2. IceTag_Standing_ < 55% and IceTag_Active_ ≥ 48%: Travel (97% probability)
3. IceTag_Standing_ ≥ 55% and IceTag_Active_ < 6%: Stand (63% probability)
4. IceTag_Standing_ ≥ 55% and IceTag_Active_ ≥ 48%: Graze (80% probability)

At the fourth split, the overall misclassification rate went down to 17% (Table 2) and there was an improvement in the identification of Graze (10% misclassification rate) at the expense of Stand (49% misclassification rate). There were no further improvements at the fifth and sixth splits. The partitions were:

1. IceTag_Standing_ < 55% and IceTag_Active_ < 48%: Lie (97% probability)
2. IceTag_Standing_ < 55% and IceTag_Active_ ≥ 48%: Travel (97% probability)
3. IceTag_Standing_ ≥ 55% and IceTag_Active_ < 6% and IceTag_Standing_ ≥ 97%: Stand (81% probability) [reduces to IceTag_Standing_ ≥ 97% and IceTag_Active_ < 6%]
4. IceTag_Standing_ ≥ 55% and IceTag_Active_ < 6% and IceTag_Standing_ < 97%: Graze (54% probability)
5. IceTag_Standing_ ≥ 55% and IceTag_Active_ ≥ 6%: Graze (80% probability)

### 3.9. Inference of GLST Activity from Lotek Collar and IceRobotics Pedometer Outputs

Inference of GLST activity by discriminant analysis of both Lotek collar and IceRobotics pedometer outputs yielded an overall misclassification rate of 14% (Table 1; Wilks’ Lambda = 0.0066, P < 0.001), and the rates according to activity were 26, 1, 16, and 6% for Graze, Lie, Stand, and Travel, respectively. The main problem with Stand was misclassification as Graze, and almost all misclassifications of Graze labeled it as Stand.

Partition analysis with three splits yielded an overall misclassification rate of 15% and separated rates of 16, 1, 30, and 17% for Graze, Lie, Stand, and Travel, respectively (Table 2). Misclassification of Graze was almost entirely as Stand, and misclassification of Stand was largely as Graze. Misclassification of Travel intervals was almost entirely as Graze. The partitions were:

1. IceTag_Standing_ < 55% and Lotek_Distance_ < 96 m: Lie (97% probability)
2. IceTag_Standing_ < 55% and Lotek_Distance_ ≥ 96 m: Travel (92% probability)
3. IceTag_Standing_ ≥ 55% and Lotek_LeftRight_ < 41: Stand (77% probability)
4. IceTag_Standing_ ≥ 55% and Lotek_LeftRight_ ≥ 41: Graze (79% probability)

Increasing the number of splits to six (involving IceTag_Standing_, Lotek_Distance_, Lotek_LeftRight_, Lotek_HeadDown_ and IceTag_Active_) resulted in an overall misclassification rate of 10%, and separated rates of 6, 1, 26, and 17% for Graze, Lie, Stand, and Travel, respectively (Table 2). The partitions were:

1. IceTag_Standing_ < 55% and Lotek_Distance_ < 96 m: Lie (97% probability)
2. IceTag_Standing_ < 55% and Lotek_Distance_ ≥ 96 m: Travel (92% probability)
3. IceTag_Standing_ ≥ 55% and Lotek_LeftRight_ < 41 and IceTag_Active_ < 6%: Stand (92% probability)
4. IceTag_Standing_ ≥ 55% and Lotek_LeftRight_ < 41 and IceTag_Active_ ≥ 6%: Graze (60% probability)
5. IceTag_Standing_ ≥ 55% and Lotek_LeftRight_ ≥ 41 and Lotek_HeadDown_ ≥ 84% and IceTag_Active_ < 6%: Stand (84% probability)
6. IceTag_Standing_ ≥ 55% and Lotek_LeftRight_ ≥ 41 and Lotek_HeadDown_ ≥ 84% and IceTag_Active_ ≥ 6%: Graze (74% probability)
7. IceTag_Standing_ ≥ 55% and Lotek_LeftRight_ ≥ 41 and Lotek_HeadDown_ < 84%: Graze (89% probability)

## 4. Discussion

Examined individually, each of the four Lotek GPS collar outputs responded clearly to GRT and GLST activities, and the effect of activity was highly significant in the non-parametric analysis of each variable. However, for our purposes, although statistical significance may be a necessary condition, it is far from being a sufficient one. For GRT activity, Lotek_LeftRight_ alone would yield a misclassification rate (by discriminant analysis) of 30%. Likewise, the misclassification rates for GRT activity based on each of Lotek_ForAft_, Lotek_HeadDown_ and Lotek_Distance_ alone would be 38, 39, and 31%, respectively. We would, therefore, advise strongly against inferring GRT activity from any single GPS collar output, even though the means of that output may differ significantly according to activity. The misclassification rate fell to approximately 16% when all collar outputs were included in the analysis, although partition analysis could achieve a misclassification rate of 17% on the basis of only two variables: Lotek_LeftRight_ and Lotek_Distance_.

For GLST activity, the case against inference on the basis of just one GPS collar output is even stronger: discriminant analysis would yield a misclassification rate of at least 50%. In the present study, even when all collar outputs were included in the analysis, the separation between Lie and Stand, by either discriminant or partition analysis, was highly problematic. In discriminant analysis the misclassification rate of Graze was greater for GLST than for GRT activity. Partition analysis avoided this problem and gave somewhat better results overall for the inference of GLST activity on the basis of GPS collar outputs. Nevertheless, GPS collar outputs were not found to be an effective basis for inference of GLST activity.

As was found for the GPS collar variables, each of the four IceRobotics IceTag pedometer outputs responded clearly to GRT and GLST activities, and the effect of activity was highly significant in the non-parametric analysis of each output. However, when considered alone, two of the outputs were very poor predictors of GRT activity, whereas the other two performed much better: IceTag_Standing_ and IceTag_Lying_ would yield misclassification rates (by discriminant analysis) of 63 and 58%, respectively; in contrast, each of IceTag_Active_ and IceTag_Steps_ (which are highly correlated) would yield a relatively low misclassification rate of 18%, although the misclassification rate for Graze would be greater than 40%. If partition analysis were used, IceTag_Active_ alone (two splits) would yield a similar overall misclassification rate, but a much more balanced error profile across the three activities. A quite highly branched partition model with five splits, involving IceTag_Active_, IceTag_Standing_ and IceTag_Steps_, was required to reduce the misclassification rate by just a few percentage points. The main problem with both the two-split and five-split models was misclassification of Rest as Graze.

For GLST activity, the misclassification rates obtained by discriminant analysis of the four respective pedometer outputs separately were 27% for IceTag_Standing_, 33% for IceTag_Active_, 59% for IceTag_Lying_, and 36% for IceTag_Steps_. Inclusion of all the outputs in the analysis yielded a substantial reduction in the misclassification rate, though each analytical method had a major weakness. Discriminant analysis misclassified 43% of Graze events as Stand, and although this problem was largely avoided by use of the four-split partition model, in this model 45% of Stand events were misclassified as Graze. Overall, pedometer outputs proved a more effective basis than GPS collar outputs for inference of GLST activity, but significant errors remained.

The finding that GRT activity could be inferred from pedometer data alone might prove useful, even when GPS data are available. Lotek collars are often operated at GPS-fix intervals much longer than 5 min, in which case a single activity cannot be assumed to continue throughout the entire interval. This requires a different analytic approach (see [19]; US data set) and different calibration equations for each combination of GPS-fix interval and activity sampling period of the motion sensors. Availability of an independent device for activity determination could be a distinct advantage in such circumstances. Since we expect more parsimonious partition models to be more robust, our recommendation would be to apply the two-split model, based on IceTag_Active_.

Combining GPS collar and pedometer data enabled achievement of a lower misclassification rate than was possible by use of either sensor alone. The lowest overall misclassification rate of GRT activity (10%) was achieved by means of a six-split partition model. However, Graze and Rest were confused in this model, with a greater proportion of Rest events misclassified as Graze than *vice versa*. Discriminant analysis yielded a broadly similar classification matrix, with the confusion between Graze and Rest being slightly more balanced. But it should be noted that implementation of partition analysis is far more computationally convenient than discriminant analysis, and is far easier to understand intuitively; we would recommend using the six-split partition model.

The level of generality is an important issue with regard to any calibration study. Some indication of this can be gleaned by comparison of the present results with those of an earlier study [19], in which data sets from the US and Israel were analyzed; here we refer only to the latter. It should be stressed at the outset that there were differences in operating conditions. First, the foraging environment in the earlier study comprised a rich herbaceous plant community with no significant presence of woody vegetation. Second, an earlier model of the Lotek GPS collar was used (without pedometers), which did not have a head-down sensor. However, the collars in both studies were configured identically, and the same methodology was used for the observational data. The data sets differed in size: 231 (previous study) versus 1,475 (present study) 5-min intervals. There were some notable differences in the shapes of the frequency distributions of collar variables according to activity. In the previous study, almost all left-right sensor counts associated with Graze and Travel were above 150, whereas in the present study, more than half were below this level (Figure 1). The fore-aft sensor yielded a fairly even frequency distribution for Travel in the previous study, in contrast to a strongly skewed distribution in the present study (Figure 1). Discriminant analysis of GRT activity yielded a misclassification rate of 14% in the previous study, with a lower rate for Graze (8%) and higher rates for Rest (17%) and Travel (22%) than those in the present study. Partition analysis of GRT yielded a misclassification rate of 12% in the previous study, with lower rates for Graze (4%) and Rest (16%) and a higher rate for Travel (22%) than in the present study. Discriminant analysis of GLST activity yielded a misclassification rate of 29% in the previous study, with the biggest problem being the misclassification of Stand as Lie, which was also a major source of error in the present study. Partition analysis of GLST activity in the previous study yielded an overall misclassification rate of 22% with a four-split model, which is clearly better than the 32% overall misclassification rate achieved with a six-split model in the present study. Considered overall, somewhat better results were obtained in the previous study than in the present one: the misclassification rates—overall and for Graze—were lower in all analyses, and there was less confusion between Graze and Rest in partition analysis. In any event, the combined use of GPS collars and pedometers in the present study yielded better results than those achieved in the earlier study.

It is possible that the presence of woody vegetation at the Hatal site (present study) may have made it more difficult to distinguish between Graze and Rest, because consumption from the shrub and tree layers slows down animal movement and may also lead to a less distinct Graze signature in terms of the motion sensor counts. We compared the rates of animal movement in the two studies, according to the distance travelled during 5-min Graze events. Means in the previous and the present studies were 24 m (n = 95) and 20 m (n = 489), respectively. Results of tests for unequal variances of the means were not significant, and those of the *t*-test assuming equal variances were not significant (*P* > 0.2). The median distances travelled during 5-min Graze events were 13 m (previous study) and 11 m (present study), and these values did not differ significantly (*P* > 0.08; Wilcoxon–Mann–Whitney test). However, differences in motion sensor responsiveness between the two models of Lotek GPS collar used cannot be ruled out.

The possible role of GPS error in the confusion between Graze and Rest was discussed in the previous study. The test that was performed then, with stationary collars, yielded relatively short distances between successive GPS location readings, even without differential correction: the 97.5 percentile, median and mean values were 9.3, 2.1 and 2.7 m, respectively. However, a similar test conducted in September 2010 with collars of the same model as that used in the present study yielded larger values: the 97.5 percentile, median and mean values were 33.6, 6.5 and 9.4 m, respectively (seven collars; ∼800 GPS positions per collar over a 70-h period; no prior screening of the data). In another test, with a stationary Lotek GPS collar [26], following differential correction, 90% of values were within 5.5 m of their mean location, and the mean distance between successive GPS locations was approximately 4.3 m. Corresponding values for our present test were 12.8 and 6.5 m, respectively. It is quite conceivable, therefore, that the Graze/Rest confusion could be reduced by using differential correction of GPS locations. Under our present operating conditions this would have presented logistic constraints, as explained earlier, but it should be considered where possible.

The equations generated by discriminant and partition analyses are dependent on the relative proportions of the various activities in the dataset. This raises the question as to whether, ideally, this balance in the calibration dataset should reflect the balance obtained in the field, as was the case, approximately, in the present study, or whether each activity should be covered by the same number of observations. We do not have a rigorous proof as to which is preferable. In a preliminary analysis, the GRT activity with the fewest records (Travel; n = 88) was used to determine the number of records that should be sampled randomly from each of the other two activity types. Partition analysis of GRT activity, based on GPS collar and pedometer outputs was then applied to the resulting dataset (n = 264). By the third split in the classification tree, the same variables were selected as in the original analysis of the complete dataset, and almost identical split thresholds were used, although the order of splitting differed. Thereafter, the two analyses diverged, but this may have been influenced by minimum cell count constraints. A more thorough investigation of this issue is warranted, but it would require a much larger dataset. This analysis does seem to indicate that more parsimonious models are more robust.

In more general terms, how easy is it to infer activity from the sensors deployed in this study? It does not seem possible to classify activity well from direct inspection of the data; calibration equations are required. The only activity that can be identified with complete confidence directly from the data is lying (from IceTag_Lying_). Travel can be separated well from other activities on the basis of Lotek_Distance_ or IceTag_Steps_, but some threshold needs to be assumed. Given that calibration equations are required, the question is how widely they can be applied. The list of factors that could conceivably influence them is long, and only a well-coordinated multi-site study can shed light on this important issue. Similarly, questions related to within- and between-animal variability and sample size await larger-scale studies.

## 5. Conclusions

In general, partition analysis performed better than discriminant analysis for inferring animal activity from outputs provided by Lotek GPS collars and/or IceRobotics IceTag pedometers. For classification of GRT activity, if partition models with only two or three splits are sought, either device alone can yield a misclassification rate of approximately 18%, and no benefit is accrued from their combined use. However, a misclassification rate of 10% can be achieved by their combined use if a more highly branched classification tree is acceptable. The primary problem in GRT classification is confusion between Graze and Rest, and if it is required to divide Rest into Lie and Stand, the use of GPS collars alone is inadequate. However, the pedometer enabled Lie activity to be inferred with high accuracy. The combination of GPS collars and pedometers yielded a misclassification rate of 10% in a six-split partition model, and misclassification of both Stand and Travel as Graze was the main source of error. If short-interval GPS fixes are not feasible, the IceRobotics IceTag pedometer alone can yield a reasonable estimation of the activity timeline of cattle on rangeland.

## Figures and Tables

**Table 1.** Frequency of observed versus predicted activity and misclassification rates obtained in the inference of animal activity, classified as GRT and GLST, by discriminant analysis of results obtained from Lotek GPS collars only, IceRobotics IceTag pedometers only, and both devices together. Elements on the upper-left to lower-right diagonal (bold) are correctly classified observations. Ideally, all observations should fall on this diagonal. G = Graze, R = Rest, T = Travel, L = Lie, S = Stand.

**Classi-fication**	**Device**	**Observed Activity**	**Predicted Activity**				**Misclassification Rate (%)**
			**G**	**R**	**S**	**T**	
GRT	GPS collar	G	**350**	136	–	3	28
		R	85	**809**	–	4	10
		T	7	1	–	**80**	9
		All					16
	Pedometer	G	**477**	3	–	9	2
		R	359	**539**	–	0	40
		T	6	0	–	**82**	7
		All					26
	Both	G	**437**	46	–	6	11
		R	100	**798**	–	0	11
		T	5	0	–	**83**	6
		All					11
GLST	GPS collar	G	**330**	46	110	3	33
		L	20	**356**	130	2	30
		S	48	154	**174**	2	54
		T	7	0	1	**80**	9
		All					36
	Pedometer	G	**270**	0	210	9	45
		L	1	**502**	5	0	1
		S	60	13	**305**	0	19
		T	6	0	0	**82**	7
		All					21
	Both	G	**363**	0	120	6	26
		L	1	**502**	5	0	1
		S	48	13	**317**	0	16
		T	5	0	0	**83**	6
		All					14

**Table 2.** Frequency of observed versus predicted activity and misclassification rates obtained in the inference of animal activity, classified as GRT and GLST, by partition (classification tree) analysis on the basis of data from the Lotek GPS collar only, the IceRobotics IceTag pedometer only, and both devices together. Elements on the upper-left to lower-right diagonal (bold) are correctly classified observations. Ideally, all observations should fall on this diagonal. G = Graze, R = Rest, T = Travel, L = Lie, S = Stand.

**Classification**	**Device**	**Number of splits**	**Observed activity**	**Predicted activity**				**Misclassification Rate (%)**
				**G**	**R**	**S**	**T**	
GRT	GPS collar	3	G	**409**	73	–	7	16
			R	167	**726**	–	5	19
			T	3	0	–	**85**	3
			All					17
		4	G	**326**	156	–	7	33
			R	37	**856**	–	5	5
			T	1	2	–	**85**	3
			All					14
	Pedometer	2	G	**429**	47	–	13	12
			R	198	**700**	–	0	22
			T	3	0	–	**85**	3
			All					18
		5	G	**428**	48	–	13	12
			R	163	**735**	–	0	18
			T	3	0	–	**85**	3
			All					15
	Both	2	G	**434**	47	–	8	11
			R	198	**700**	–	0	22
			T	3	0	–	**85**	3
			All					17
		6	G	**451**	30	–	8	8
			R	110	**788**	–	0	12
			T	3	0	–	**85**	3
			All					10
GSLT	GPS collar	6	G	**409**	13	60	7	16
			L	57	**330**	118	3	35
			S	106	102	**168**	2	56
			T	3	0	0	**85**	3
			All					32
	Pedometer	3	G	**309**	0	178	2	37
			L	1	**505**	2	0	1
			S	59	16	**303**	0	20
			T	15	0	0	**73**	17
			All					19
	Both	3	G	**411**	0	76	2	16
			L	2	**501**	1	4	1
			S	97	16	**265**	0	30
			T	12	0	3	**73**	17
			All					15
		6	G	**458**	0	29	2	6
			L	3	**501**	0	4	1
			S	84	16	**278**	0	26
			T	15	0	0	**73**	17
			All					10

## Abbreviations (Definition, Unit):

GLST: (Classification of activity as Graze, Lie, Stand, Travel, −)
GRT: (Classification of activity as Graze, Rest, Travel, −)
IceTag_Active_: (IceRobotics IceTag pedometer percentage of time interval in active state, %)
IceTag_Lying_: (IceRobotics IceTag pedometer percentage of time interval in lying state, %)
IceTag_Standing_: (IceRobotics IceTag pedometer percentage of time interval in standing state, %)
IceTag_Steps_: (IceRobotics IceTag pedometer number of steps taken during time interval, −)
Lotek_Distance_: (Lotek GPS collar straight-line distance between consecutive coordinates at 5-min intervals, m)
Lotek_ForAft_: (Lotek GPS collar fore-aft motion sensor count (value between 0 and 255, −)
Lotek_HeadDown_: (Lotek GPS collar Head Down indicator, %)
Lotek_LeftRight_: (Lotek GPS collar left-right motion sensor count (value between 0 and 255, −)
